# Editorial: mHealth and smartphone apps in patient follow-up

**DOI:** 10.3389/fmedt.2025.1644384

**Published:** 2025-07-07

**Authors:** Uffe Kock Wiil

**Affiliations:** SDU Health Informatics and Technology, the Maersk Mc-Kinney Moller Institute, University of Southern Denmark, Odense, Denmark

**Keywords:** mobile health (mHealth), smartphone, patient follow-up, clinical decision aid, treatment adherance

**Editorial on the Research Topic**
mHealth and smartphone apps in patient follow-up

Mobile health (mHealth) is an approach that uses telecommunication devices such as personal digital assistants, smartphones, laptops, etc. to provide health care and information. In recent years, mobile health technology has been used for various purposes, including (1) supporting clinical diagnosis and/or decision-making, (2) acting as standalone digital therapeutics, (3) improving access to health services, (4) adherence to treatment, (5) management of chronic diseases, (6) education, monitoring, and communication with the patient, and (7) reduction in the burden of diseases caused by poverty and patients follow up [i.e., (1–3)].

Mobile health will play an important role in various situations related to patient follow-up in the future [i.e., (4, 5)]. Mobile health technology can facilitate the process of follow-up and patient management to improve the quality of patient care. Examples include children's vaccinations, monitoring patients with chronic diseases, observing patients’ behavioral changes, and identifying at-risk patients that need care and referral services. Various communication schemes such as SMS, video messages (MMS), phone calls, and push notifications can be used when developing novel mobile applications for patient follow-up.

The main goal of this Research Topic is to focus on challenges, limitations, opportunities, and potential impact of mobile healthcare technology on patient follow-up from practical, experimental, or theoretical perspectives. It is important that the development of mobile health technology takes a holistic and interdisciplinary approach involving diverse stakeholders (i.e., patients and their relatives, healthcare professionals, hospital administrators, healthcare providers, government officials, etc.) and emphasize how these stakeholders benefit from the proposed mobile health solutions.

We received 29 submissions for this Research Topic. 18 quality papers were accepted involving 93 authors from 11 countries including South Korea, China, Belgium, Italy, Iran, Pakistan, Malaysia, Mexico, Nigeria, Lithuania, and the United States. The 18 accepted papers consist of 11 original research papers, 5 systematic reviews, and 2 brief research reports. We provide a short introduction to each accepted paper below.

Yu et al. conducted a systematic literature review and meta-analysis of randomized controlled trials focusing on the technological functionality and system architecture of mobile health interventions for diabetes management. 41 out of 3,911 identified studies published prior to August 2024 were selected for the analysis which confirmed that mobile health applications are beneficial in managing diabetes.

Qin et al. introduced an intelligent question-answering system designed to deliver personalized medical information to diabetic patients. The system demonstrated the effectiveness in integrating large language models with knowledge graphs to provide more accurate and contextually relevant medical guidance, addressing the limitations of traditional healthcare systems in handling complex medical queries.

Li et al. conducted a systematic literature review that explored the impact of telemedicine in chronic patients from diverse socioeconomic contexts. A total of 35 out of 10,755 identified studies published prior to November 2024 were selected for analysis. Four themes that can improve future telemedicine for patients with chronic diseases were synthesized: reminders and supervisors, access to knowledge, transition in medical treatment mode, and emotional support platform.

Heidari et al. developed an mHealth solution to support a lifestyle modification intervention among pregnant Iranian women with hypertension based on self-determination theory. The developed solution was evaluated on 60 Iranian women (of which 30 were in the control group) in a study running from 2021–2023. The intervention demonstrated improvements in lifestyle factors and self-determination constructs.

Kyriazakos et al. conducted a systematic literature review focusing on clinical outcomes of Software as a Medical Device (SaMD) and mHealth applications in the areas of remote patient monitoring (RPM) and digital therapeutics (DTx). 28 out of 629 identified studies published prior to April 2024 were selected. The review demonstrated the significant potential in revolutionizing chronic disease management through RPM and DTx exemplified by solutions like Healthentia.

Sattar et al. assessed online pharmacy applications in India by employing the Mobile Application Rating Scale (MARS). The authors identified 40 online Indian pharmacy apps in Google Play Store and App Store of which 13 were chosen and evaluated using MARS. The MARS evaluation demonstrated significant positive associations across the four quality scales. The mean rating of the 13 apps fell between the range of 3.11 to 4.32 on a 5-point scale.

Hosseinnia et al. conducted a systematic literature review focusing on applications for the management of Attention Deficit Hyperactivity Disorder (ADHD) that have been evaluated in various types of trials. A total of 14 out of 281 identified studies published prior to May 2024 were included. The applications targeted different facets of ADHD, including symptom monitoring, medication adherence, cognitive training, specific functioning, and behavior management.

Sun et al. explored how doctors' social media behavior affects patient adherence and treatment outcome. The study showed that doctors posting professional knowledge content on social media positively impacted patient adherence and treatment effectiveness. In contrast, doctors sharing personal life-related content on social media were associated with lower patient adherence and poorer treatment outcome.

The study by Qui and Zhou assessed the quality of heatstroke videos on TikTok. 90 out of the top 100 heatstroke-related videos were examined focusing on their characteristics, quality, and the content they conveyed. The study showed that the quality of information provided in heatstroke-related videos on TikTok is generally inadequate and requires significant improvement. In addition, such content should be subject to government review to ensure its accuracy and reliability.

Fei et al. analyzed the continuance intention of digital health resources. The study used cross-sectional surveys and structural equation modeling analysis to explore factors influencing user willingness to continue using digital health resources. In summary, the keys to solving the problem of low continuance intention are improving the quality and service level of digital health resources and promoting users' value co-creation behavior.

Meng and Guo examined the factors influencing users' intention to continue using mobile medical apps within the framework of the Unified Theory of Acceptance and Use of Technology model. Through a combination of questionnaire surveys and interviews, the study finds that doctor-patient trust, performance expectancy, social influence, and facilitating conditions significantly impact users' intention to utilize mobile medical apps.

Zhong et al. explored the relationship between information overload and overuse of medical services in mHealth applications using the health belief model. Data was collected from 1,494 respondents using a structured questionnaire. The study found that information overload significantly affected users' perceived severity, susceptibility, treatment benefits, barriers, self-efficacy, and action cues, and subsequently affected overuse of health care services.

Liu et al. conducted a systematic literature review focusing on the efficacy of telemedicine intervention in self-management of patients with type 2 diabetes. 17 out of 4,171 identified studies published prior to December 2023 were selected for the analysis. The review confirmed that telemedicine interventions may assist patients with type 2 diabetes in enhancing their self-management and improving their blood glucose levels.

Ortíz et al. performed a usability evaluation of the educational website “I understand my diabetes” (“Entiendo mi diabetes”) for Mexican patients with type 2 diabetes attending primary care clinics. A cross-sectional study was done in 110 patients with type 2 diabetes from two family medicine clinics. The study found that the educational website had an adequate usability evaluation by patients, and they also considered it very useful for diabetes care.

Han and Li studied the use of Online Follow-Up Services (OFUS) as a supplement to ordinary hospital care available through online health communities (OHC). A dataset including 3,672 doctors in a leading OHC in China was analyzed using the elaboration likelihood model. The results show that both doctors' knowledge contributions and patient feedback positively influence patients' effective use of OFUS.

Babatunde et al. reported on the development and evaluation of an educational and monitoring mobile application for pregnant women in Nigeria. A method of user-centered design was employed involving pregnant women attending antenatal clinics in Oyo State, Nigeria. The study demonstrated that mHealth applications have the potential to increase access to prenatal information and services in Nigeria and may reduce maternal and childhood mortality.

Zhao et al. explored the potential of a computer vision-based method to perform sit-to-stand test for home-based self-assessment of physical condition in advanced knee osteoarthritis (KOA) patients. 104 KOA patients were enrolled in the study to evaluate their physical function and joint stiffness. The study demonstrated the potential for low-cost and user-friendly self-assessment of KOA patients with varying stiffness levels and functional limitations utilizing smartphones.

Kasputytė et al. analyzed the association between the behavior of cancer patients, measured using passively and continuously generated data streams from smartphone sensors, and perceived fear of COVID-19 and COVID-19 vaccination status. 202 cancer patients were included in the study, which showed that the COVID-19 pandemic continues to have an impact on the behavior of cancer patients even after the termination of the global pandemic.

While the included studies demonstrate important and relevant application areas of mobile health technology in patient follow-up, they also demonstrate some limitations that need to be addressed in future studies. Overall, it is important to focus on aspects that mature the technology and demonstrate its full potential and effects on patient care and various other important perspectives to facilitate future large-scale implementation and user adaptation. This includes but is not limited to:

• Ensure proper user involvement strategies during development (i.e., using a codesign method) as well as rigorous usability testing and evaluation to enhance user acceptance and usability using appropriate scientific methods.
• Demonstrate various effects and investigate different aspects of the developed technology, i.e., using a health technology assessment method that through a multidisciplinary process summarizes information about the medical, social, economic, ethical, and legal (i.e., GDPR and MDR in the European Union) issues in a systematic, transparent, unbiased, and robust manner to uncover strengths and limitations of the developed technology [i.e., (6)].

We hope to see an increased focus to bring mobile health technology into real clinical practice to fully unlock its potential to improve future health outcomes and quality of life.

## References

[1] Pedersen SS Andersen CM Ahm R Skovbakke SJ Kok R Helmark C Efficacy and cost-effectiveness of a therapist-assisted web-based intervention for depression and anxiety in patients with ischemic heart disease attending cardiac rehabilitation [eMindYourHeart trial]: a randomized controlled trial protocol. BMC Cardiovasc Disord. (2021) 21:20. 10.1186/s12872-020-01801-w 33413109 PMC7788554

[2] Baskaran R Møller K Wiil UK Brabrand M. Using facial landmark detection on thermal images as a novel prognostic tool for emergency departments. Front Artif Intell. (2022) 5:815333. 10.3389/frai.2022.815333 35647532 PMC9137349

[3] Werenberg Sigvardt M Friis Lindegaard R Wiil UK Peimankar A Ebrahimi A. A. Patient satisfaction with digital health solutions for depression treatment: a scoping review. *Intelligent Health Systems - From Technology to Data and Knowledge: Proceedings of MIE 2025. Studies in Health Technology and Informatics*, Vol. 327. IOS Press (2025). p.1145–9. 10.3233/SHTI250569 40380674

[4] Christie SA Mbianyor M Oke R Dissak-Delon F Yakue F Essomba F Mobile health follow-up screening to risk stratify patients in need of further care in a low resource setting: results from a prospective multisite implementation study. J Trauma Acute Care Surg. (2023) 95(5):699–705. 10.1097/TA.0000000000003991 37876247 PMC10605648

[5] Gómez-González MA Cordero Tous N De la Cruz Sabido J Sánchez Corral C Lechuga Carrasco B López-Vicente M Following up patients with chronic pain using a mobile app with a support center: unicenter prospective study. JMIR Hum Factors. (2025) 12:e60160. 10.2196/60160 39844381 PMC11776344

[6] EU Health Technology Assessment Regulation (EU HTAR 2021/2282). Available at: https://health.ec.europa.eu/health-technology-assessment/overview_en (Accessed July 01, 2025).

